# Brawn and Brain

**Published:** 1893-03

**Authors:** 


### BRAWN AND BRAIN.

Not to the opulent who hoard
  Their ample store to selfish aims,
Not to the wielders of the sword
  Who smite their way on bloody plains;
Nor yet the cunning or untoward,
  Is the world debtor for its gains ;
But to the strong on land and main,
Who make advance with brawn and brain.

